# Molecular and epidemiologic characterization of the diphtheria outbreak in Venezuela

**DOI:** 10.1038/s41598-021-85957-1

**Published:** 2021-03-18

**Authors:** Ricardo A. Strauss, Laura Herrera-Leon, Ana C. Guillén, Julio S. Castro, Eva Lorenz, Ana Carvajal, Elizabeth Hernandez, Trina Navas, Silvana Vielma, Neiris Lopez, Maria G. Lopez, Lisbeth Aurenty, Valeria Navas, Maria A. Rosas, Tatiana Drummond, José G. Martínez, Erick Hernández, Francis Bertuglia, Omaira Andrade, Jaime Torres, Jürgen May, Silvia Herrera-Leon, Daniel Eibach

**Affiliations:** 1 Bernhard Nocht Institute for Tropical Medicine, Hamburg, Germany; 2 Instituto de Salud Carlos III, Madrid, Spain; 3 Instituto de Medicina Tropical, Universidad Central de Venezuela, Caracas, Venezuela; 4 Hospital José María Vargas, Caracas, Venezuela; 5 Hospital Universitario de Caracas, Caracas, Venezuela; 6 Ciudad Hospitalaria Dr Henrique Tejera, Carabobo, Venezuela; 7 Centro Clinico-Materno Leopoldo Aguerrevere, Caracas, Venezuela; 8 Hospital Universitario de Los Andes, Mérida, Venezuela; 9 Hospital Universitario de Maracaibo, Maracaibo, Venezuela; 10 Hospital de Niños José Manuel de Los Ríos, Caracas, Venezuela; 11 Hospital General Los Magallanes de Catia, Caracas, Venezuela; 12 Hospital Raúl Leoni, Ciudad Guayana, Venezuela

**Keywords:** Infectious diseases, Microbiology, Health care

## Abstract

In 2016, Venezuela faced a large diphtheria outbreak that extended until 2019. Nasopharyngeal or oropharyngeal samples were prospectively collected from 51 suspected cases and retrospective data from 348 clinical records was retrieved from 14 hospitals between November 2017 and November 2018. Confirmed pathogenic *Corynebactrium* isolates were biotyped. Multilocus Sequence Typing (MLST) was performed followed by next-generation-based core genome-MLST and minimum spanning trees were generated. Subjects between 10 and 19 years of age were mostly affected (n = 95; 27.3%). Case fatality rates (CFR) were higher in males (19.4%), as compared to females (15.8%). The highest CFR (31.1%) was observed among those under 5, followed by the 40 to 49 age-group (25.0%). Nine samples corresponded to *C. diphtheriae* and 1 to *C. ulcerans.* Two Sequencing Types (ST), ST174 and ST697 (the latter not previously described) were identified among the eight *C. diphtheriae* isolates from Carabobo state. Cg-MLST revealed only one cluster also from Carabobo. The Whole Genome Sequencing analysis revealed that the outbreak seemed to be caused by different strains with *C. diphtheriae* and *C. ulcerans* coexisting. The reemergence and length of this outbreak suggest vaccination coverage problems and an inadequate control strategy.

## Introduction

Diphtheria most frequently presents as an upper respiratory infection predominantly caused by toxigenic strains of the gram-positive bacilli, *Corynebacterium diphtheriae.* Symptoms include sore throat, mild fever, and a gray-white pseudomembrane on the tonsils, larynx or pharynx. In the industrialized world, the infection declined to only sporadic cases after the introduction of the toxoid vaccine in 1923 and massive immunization in 1940–1950^1^. However, despite the implementation of the Expanded Immunization Program by the World Health Organization in 1974, Diphtheria remains endemic in several developing countries. Its re-emergence is usually associated with the breakdown of health systems which struggle to reach adequate vaccination coverage^2^. This was observed in the former USSR in the early 1990s with around 150,000 older children and adults affected^3^. More recently, clusters and outbreaks have been reported from South Africa, Bangladesh, Laos and Yemen^4–7^.

Between July and November 2016, Venezuelan doctors at small urban health centers reported an unusual increase of “atypical pharyngitis” cases, presenting with pharyngeal lesions resembling diphtheria*.* The last previous case of the disease in the country was officially reported in 1992^8^.

The first cases were reported in July 2016 in the southern-border Bolivar state and progressively spread throughout all states and to neighboring countries, such as Colombia^9^. As of epidemic week 48 of 2019, a total of 3,033 suspected cases with peaks in the years 2017 and 2018 and an official reported Case Fatality Rate (CFR) of 9% were reported^10^. The accuracy of this data has been subject of debate by several local non-governmental health organizations that indicated a higher incidence than the reported as well as important delays on the official publication of epidemiological data and reporting inaccuracies^11^.

The available data provided by PAHO (Panamerican Health Organisation) is aggregated on a national level and only represented by case definition and survival status. This study aims at characterizing the molecular epidemiology of the diphtheria outbreak in Venezuela by gathering retrospective and current demographic data of cases as well as available current samples obtained directly from hospitals located in the most affected regions.

## Methods

### Data collection and study sites

Between December 2017 and December 2018 (one of the outbreak peak periods^10^) nasopharyngeal (NP) or oropharyngeal (OP) swabs from suspected cases were collected at 14 hospitals across the most affected states: 6 hospitals in the Capital District, 2 hospitals in Carabobo, and 1 hospital in each of the following states: Aragua, Bolivar, Mérida, Anzoátegui, Lara and Zulia. Demographic information was retrieved from direct interviews with the patients from whom samples were taken (Tables 1 and 3).

**Table 1:** Descriptive characteristics of the study participants (N = 51) recruited between December 2017 and December 2018 in Venezuela.

Characteristic	N	n (%)
**Gender**	51^a^	
Female		30 (65.2)
Male		16 (34.8)
**Age (years)**	51^b^	
< 5		10 (22.7)
5–9		6 (13.6)
10–19		18 (40.9)
20–29		4 (9.1)
30–39		2 (4.5)
40–49		2 (4.5)
≥ 50		2 (4.5)
**State**	51^c^	
Carabobo		16 (38.1)
Bolivar		6 (14.3)
Zulia		1 (2.4)
Merida		5 (11.9)
Distrito Capital		11 (26.2)
Miranda		1 (2.4)
Vargas		1 (2.4)
Aragua		1 (2.4)
**Membrane localization**	51^d^	
Pharyngeal		28 (87.5)
Extra pharyngeal		4 (12.5)

*N, n* sample size.

^a^Missing gender information in 5 patients.

^b^Missing age information in 7 patients.

^c^Missing state information in 9 patients.

^d^Missing membrane localization information in 19 patients.

In parallel, medical records from cases diagnosed as “Diphtheria”, “suspected Diphtheria”, “Atypical Pharyngotonsilitis” and “Atypical Pharyngitis” were retrospectively reviewed at the study hospitals from June 2016 to July 2019. Relevant demographic data was retrieved when available. Informed consent was obtained from all subjects or, if subjects were under 18, from a parent and/or legal guardian. Ethical approval for the investigation was sought and granted at the Central University of Venezuela. Also, all partners involved signed a material transfer agreement for this investigation (Table 2 and figures).

**Table 2:** Descriptive characteristics of the study population in the diphtheria outbreak (N = 348) reported between June 2016 and July 2019 in Venezuela (retrospectively retrieved from study hospital records).

Characteristic	N	n (%)	N ^a^	n (%)^a^	# deaths^a^	CFR [95% CI]^a^
**Gender**	348^b^		262			
Female		184 (52.87)		133 (50.76)	21	15.79 [10.57–22.93]
Male		164 (47.13)		129 (49.24)	25	19.38 [13.49–27.05]
**Age (years)**	348^c^		261^d^			
< 5		77 (22.13)		45 (17.24)	14	31.11 [19.53–45.66]
5–9		68 (19.54)		46 (17.62)	10	21.74 [12.26–35.57]
10–19		95 (27.30)		75 (28.74)	6	8.00 [3.72–16.37]
20–29		27 (7.76)		23 (8.81)	2	8.70 [2.42–26.80]
30–39		31 (8.91)		28 (10.73)	3	10.71 [3.71–27.20]
40–49		31 (8.91)		28 (10.73)	7	25.00 [12.68–43.36]
50–59		7 (2.01)		7 (2.68)	1	14.29 [2.57–51.31]
≥ 60		12 (3.45)		9 (3.45)	2	22.22 [6.32–54.74]
**State**	346^e^		262			
Carabobo		206 (59.54)		188 (71.76)	36	19.15 [14.16–25.37]
Bolivar		62 (16.47)		4 (1.53)	0	0.00 [0.00–48.99)]
Zulia		37 (10.69)		37 (14.12)	2	5.41 [1.50–17.70]
Merida		20 (5.49)		18 (6.87)	3	16.67 [5.84–39.22]
Distrito Capital		15 (4.05)		7 (2.67)	3	42.86 [15.82–74.95]
Miranda		9 (2.60)		7 (2.67)	1	14.29 [2.57–51.31]
Monagas		2 (0.58)		0 (0.00)	0	–
Aragua		2 (0.58)		1 (0.38)	1	100.00 [20.65–100.00)]
**Case definition**	349		262			
Suspected		314 (89.97)		237 (90.46)	40	16.88 [12.65–22.17]
Confirmed		35 (10.03)		25 (9.54)	6	24.00 [11.50–43.43]

*N, n* sample size; *CFR* case fatality rate; *CI* confidence interval.

^a^Excluding observations with missing survival status.

^b^Missing gender information in 1 patient.

^c^Missing age information in 1 patient.

^d^Missing age information in 1 patient.

^e^Missing state information in 3 patients.

### Case definitions

A suspected case was defined as any person with upper respiratory tract infection with either tonsillitis, laryngitis, or nasopharyngitis or a combination of them and either pseudomembrane or membrane or both. Confirmed cases included all suspected cases of a confirmed case with laboratory isolation of *C. diphtheriae*.

### Laboratory procedures

#### Isolation, identification and antimicrobial testing

The specimens were inoculated onto blood agar, Hoyle’s Tellurite and Tinsdale’s medium. Suspected isolates were identified and tested for toxigenicity by Polymerase Chain Reaction (PCR)^12^. Confirmed *C. diphtheriae* isolates were biotyped using the API Coryne system (Biomerieux). Toxigenicity was confirmed by the modified ELEK test. Resistance testing was performed with Etests (bioMerieux) and MICs were interpreted according to Clinical Laboratory Standards Institute (CLSI) recommended breakpoints.

### Sequencing and data analysis

For DNA extraction, we used isolates from blood agar plates. We extracted genomic DNA by using a modified protocol of the Qiamp DNA Mini Kit (Qiagen, Germany).

Routine Multilocus Sequence Typing (MLST) was performed by PCR of the 7 target regions atpA, dnaE, dnaK, fusA, leuA, odhA, and rpoB by using a previously described protocol^13^^.^ We included the modified primers described by Both et al.^14^ to be able to amplify dnaE and dnaK of C. ulcerans. Sequences were analysed using SeqMan Pro (Ver.Seqman;DNASTAR), Madison, WI (https://www.dnastar.com/blog/category/publications/). Allelic profiles and sequence type (ST) designations for each studied strain were obtained by submitting the generated DNA alleles to the PubMLST database curated by the Pasteur Institute Paris (http://pubmlst.org/cdiphtheriae/).

Whole-genome libraries for Next-generation Sequencing (NGS) were prepared with the Nextera XT kit (Illumina, San Diego, CA, USA), and sequencing was performed with 2 × 250-bp paired-end reads on the Illumina MiSeq. Quality control of NGS sequencing run was performed by the Bioinformatic Unit using FastQC v0.11.8^15^ and Trimmomatic v.0.33^16^.

We generated a *C. diphtheria* core genome-MLST (cg-MLST) scheme, defining specific target loci for whole-genome sequencing data typing, by using SeqSphere + target definer tool (Ridom, Munster, Germany) with default options. As reference, the genome of strain NC 13129 from the National Center for Biotechnology Information (NCBI) (accession number no. BX248353.1/NC_002935.2) was used. All 23 complete *C. diphtheriae* available from NCBI as query sequences (accession nos. NC_016782.1, NC_016783.1, NC_016785.1, NC_016786.1, NC_016787.1, NC_016788.1, NC_016789.1, NC_016790.1, NC_016799.1, NC_016800.1, NC_016801.1, NC_016802.1, NZ_CP018331.1, NZ_CP020410.2, NZ_CP025209.1, NZ_CP029644.1, LT_990688.1, NZ_LR134538.1, NZ_LR134537.1, NZ_CP039523.1, CP038789.1, NZ_CP039522.1, and NZ_CP039523.1) were included. The complete genome of strain NC_CPO25209.1 was used to exclude genes that are horizontally transferred to prevent that such genes are part of a cgMLST typing scheme^17^.

The resulting cg-MLST scheme consisted of 1441 target loci^17^. The accessory scheme contains 640 genes that are not homologous and do not have invalid start/stop codons in the seed genome, but overlap with other genes, do not appear in all query genomes or have invalid stop codons in 80% or more of the query genomes. By convention, those genes are not used for cgMLST. However, they can be used in addition to increase the discriminatory power if the resolution of cgMLST is not high enough.

We performed next-generation-based cg-MLST with reference-based alignments after read trimming and assembling by using Unicycler v.0.4.6^18^, Quast v4.1^19^, Kmerfinder v.3.1^20^, Snippy v.4.4.0^21^, BamUtil v.1.0.13^22^, and Picard WGSMetrics v1.140 by the Bioinformatic Unit at the Instituto de Salud Carlos III. We performed in silico cg-MLST by using the generate cg-MLST or extended cg-MLST scheme of 1.441 or 2.176 target loci. The combination of all alleles in each strain formed an allelic profile that was used to generate minimum spanning trees (MST). Alleles with missing values in at least one sample or samples with 10% of distance columns were excluded from the comparison table. A cluster was defined as a group of closely related cg-MLST-analysed isolates differing by ≤ 5 alleles and subclusters with the same similarity threshold but after extended cg-MLST.

Isolation and identification were conducted at the microbiology laboratory at the *Centro Clínico Materno Leopoldo Aguerrevere* in Caracas, Venezuela. All the subsequent laboratory procedures were carried out at the *Centro Nacional de Microbiología, Instituto de Salud Carlos III* located in Madrid, Spain.

Statistical analyses were conducted using R version 3.4.2. Categorical variables are reported as numbers and percentages of patients. Percentages are based on all observations, excluding missing values. Case Fatality Rates (CFR) were calculated along with 95% confidence intervals. CFRs are calculated excluding those in which the survival status is missing. Histograms were used to display the distribution of cases over the outbreak period by case status, survival status and state. The map has been generated using R version 3.6.2 (https://www.r-project.org, packages: ggspatial, rnaturalearth).

All methods including patients recruitment, sample collection, packaging and shipping of samples as well as bacteriology and molecular procedures were carried out in accordance with updated relevant guidelines and corresponding regulations. This study was reviewed and granted by an ad hoc ethics committee from the Tropical Medicine Institute at the *Universidad Central de Venezuela*.

## Results

### Characteristics of the patients

51 cases were recruited and a corresponding number of NP and OP samples were taken depending on the membrane localization. In our cohort, females were more frequently affected (65.2%; n = 30) than males (34.8%; n = 16) and young subjects with ages between 10 and 19 (40.9%; n = 18) as well. Most of the cases were found in hospitals from the central and densely populated states of Carabobo (38.1%; n = 16) and Capital District (26.2%; n = 11). Pharyngeal localization of the pseudomembrane was predominant (87.5%; n = 28) in comparison to extra-pharyngeal (mostly laryngeal) pseudomembranes (12.5%; n = 4) (Table 1).

As for the retrospective data, more females (52.0%; n = 184) than males were affected yet being the CFR 19.4% in males and 15.8% in females Young individuals were the most frequently affected with a majority (27.3%; n = 95) of cases between ages of 10 to 19 years of age, followed by children under 5 years (22.1%; n = 77) and then by children between 5 and 10 years old (19.5%; n = 68). The highest CFR (31.1%) was observed among those under 5, followed by the 40 and 49 years age-group (25.0%). The state with most cases was Carabobo with 206 (59.5%) cases; a highly populated coastal-central state located 167 km to the west of the capital. Following, Bolivar and Zulia accounted for 62 (16.5%) and 37 (10.7%) cases respectively. The capital ranked fifth place in number of cases, although it registered the highest CFR of 42.9%, followed by Carabobo, Mérida and Miranda presenting CFRs of 19.2%, 16.7% and 14.3% respectively. As for Aragua state, the only case recorded in this data died (Table 2).

**Figure 1:**
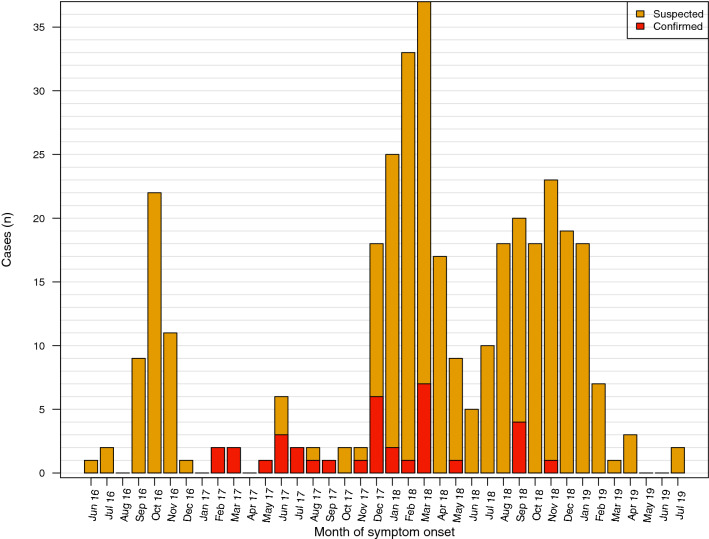
Distribution of suspected and confirmed diphtheria cases by month, Venezuela, June 2016 and July 2019 (N = 349).

**Figure 2:**
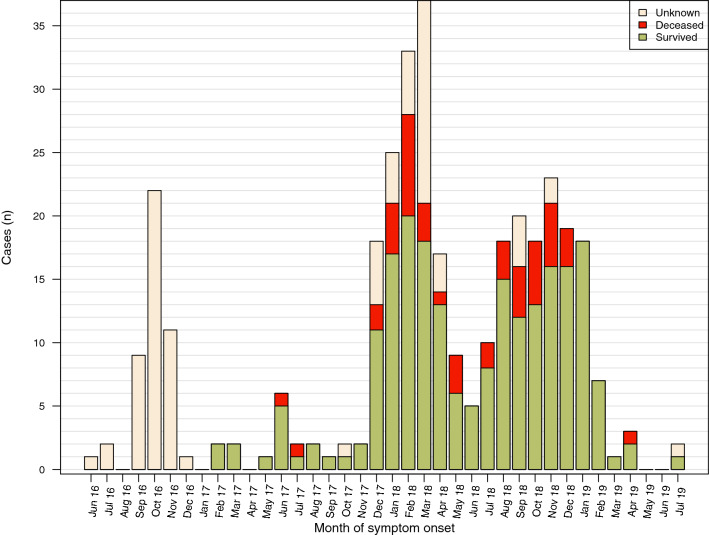
Distribution of deceased and survived diphtheria cases by month, Venezuela, June 2016 and July 2019 (N = 349 of whom 262 had information on survival status available).

In accordance with PAHO’s epidemiological updates, the case distribution based on retrieved data from the study hospitals, shows three main peaks: the first one occurring in October 2016, the second between December 2017 and April 2018, and a third wave between July 2018 and February 2019 (Fig. 1). The curve shows the low laboratory confirmation capacity. Figure 2 shows a proportional CFR which increases during the periods of highest incidence.

**Figure 3:**
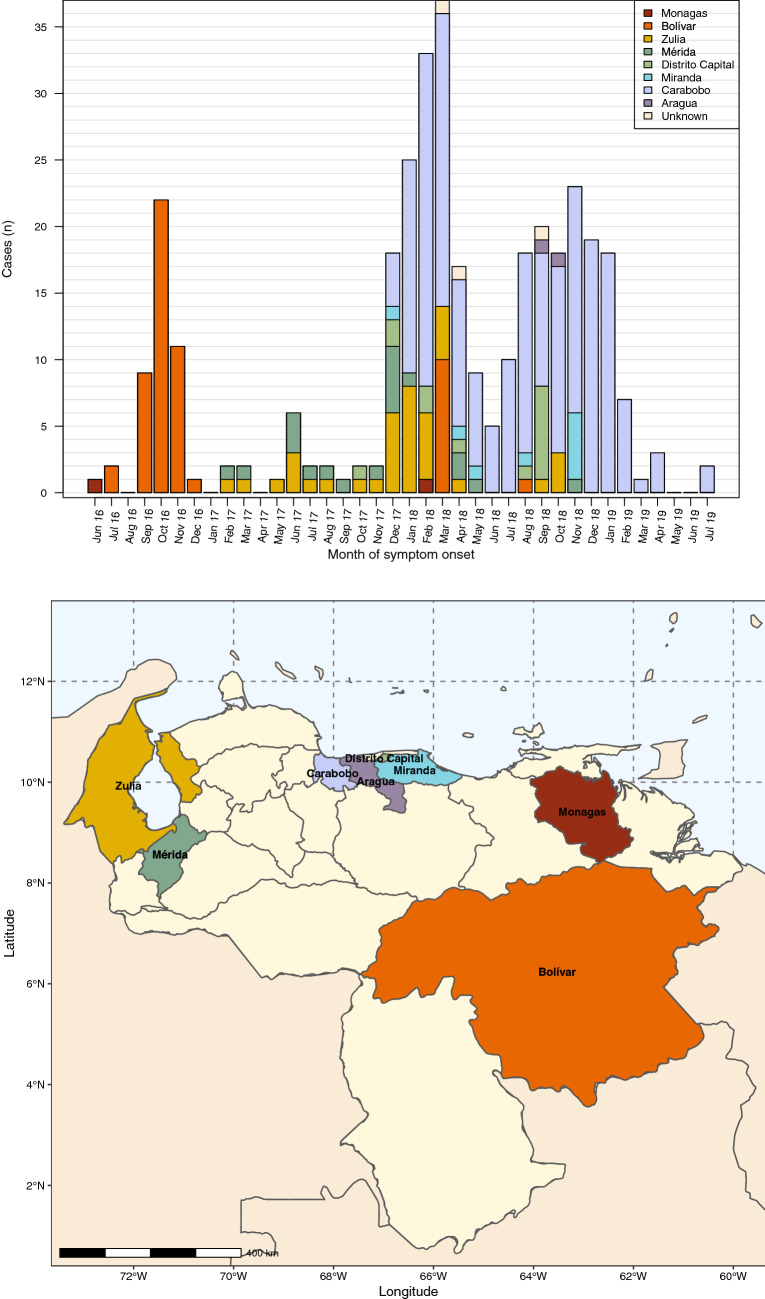
(**a**) Distribution of origin of diphtheria cases by epidemiological month of onset of symptoms, Venezuela, June 2016 and July 2019 (N = 349 of whom 346 had information on state available). **(b)** Map of Venezuela and affected regions.

**Figure 4:**
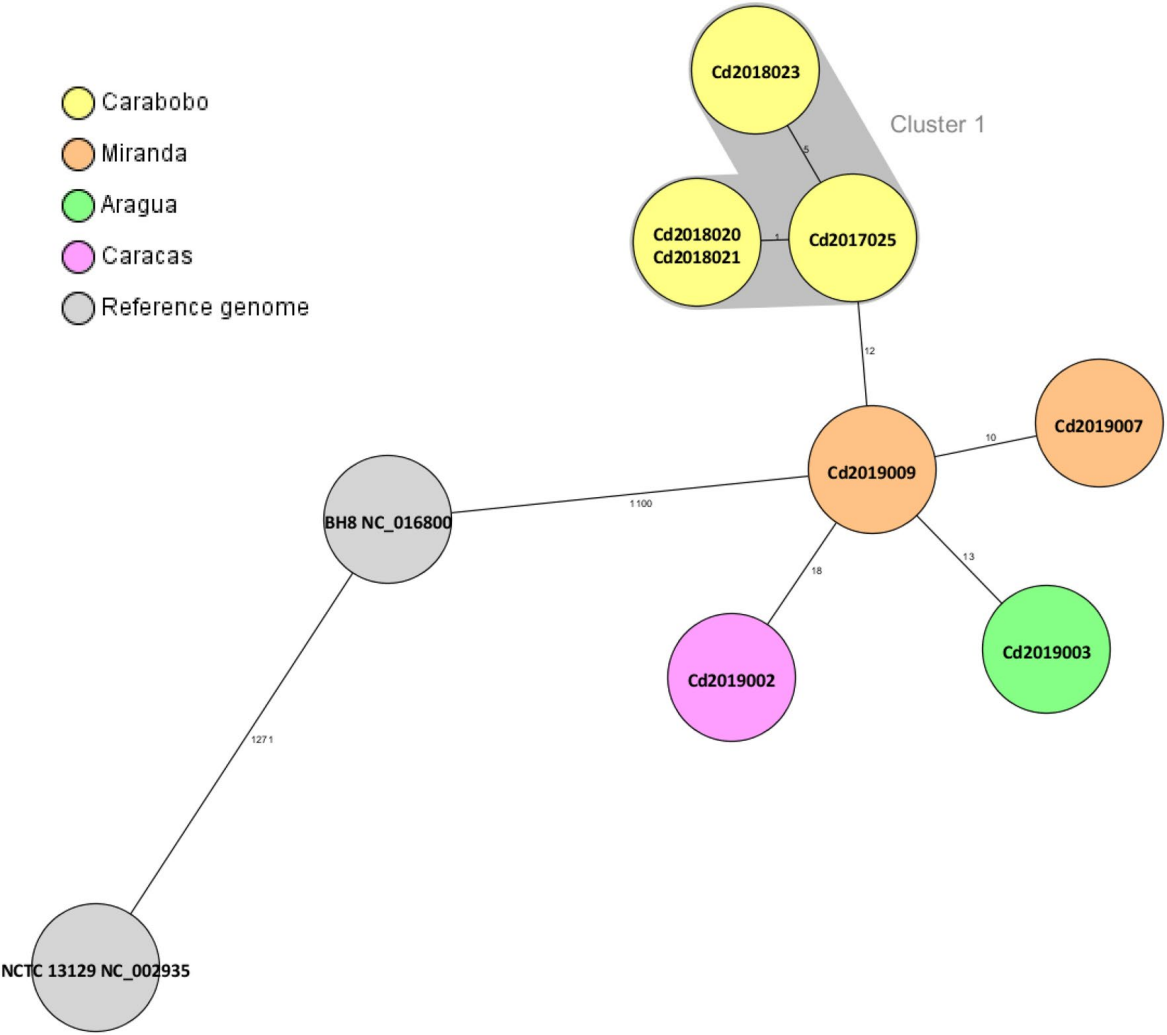
Minimum-spanning tree of core genome multilocus sequence typing with 1423 targets of the toxigenic *Corynebacterium diphtheriae* isolates from Venezuela. Allele distances between isolates are indicated, cluster with allele difference ≤ 5 is shaded in pink, and the reference genomes are shown in grey (only the reference genome used as seed and the closest reference genome to Venezuelan isolates are shown).

Figure 3 shows the different states according to the number of cases during the outbreak. The distribution of cases retrospectively retrieved shows a peak of cases in Bolivar state which shares borders with Brazil. Following this peak, cases were reported from the western states Zulia and Merida, which share a border with Colombia. Here, the number of cases remained low. An increasing number of cases was registered from December 2017 onwards in the densely populated coastal-central area, in which Carabobo state presented the highest disease burden by the end of 2019 (Figs. 3 and 4).

### Microbiological characteristics

Among 51 samples collected for the present study, *C. diphtheriae* was detected in 9 (18%) cases. One case was positive by PCR but the culture was negative. All *C. diphtheriae* isolates corresponded to biotype *mitis*. *C. ulcerans* was isolated in one (2%) case. All isolates resulted in toxigenic strains confirmed by PCR and ELEK (Table 3).

**Table 3:** Characteristics of the C*orynebacterium* isolates.

N°	ID	Sex	Age	Diagnosis	State	Specimen	Pathogen (biotype)	Elek	Tox Gen PCR	ST
1	2,017,024	F	66	Respiratory diphtheria	Mérida	*Corynebacterium diphtheriae*	mitis	Yes	Yes	ND
2	2,017,025	F	10	Respiratory diphtheria	Carabobo	*Corynebacterium diphtheriae*	mitis	Yes	Yes	697
3	2,018,021	F	12	Respiratory diphtheria	Carabobo	*Corynebacterium diphtheriae*	mitis	Yes	Yes	697
4	2,018,020	F	3	Respiratory diphtheria	Carabobo	*Corynebacterium diphtheriae*	mitis	Yes	Yes	697
5	2,018,023	M	11	Respiratory diphtheria	Carabobo	*Corynebacterium diphtheriae*	mitis	Yes	Yes	697
6	2,019,002	F	61	Respiratory diphtheria	Distrito Capital	*Corynebacterium diphtheriae*	mitis	Yes	Yes	174
7	2,019,006	F	17	Respiratory diphtheria	Distrito Capital	*Corynebacterium* *ulcerans*	NA	Yes	Yes	702
8	2,019,003	M	44	Respiratory diphtheria	Aragua	*Corynebacterium diphtheriae*	mitis	Yes	Yes	174
9	2,019,007	F	38	Respiratory diphtheria	Miranda	*Corynebacterium diphtheriae*	mitis	Yes	Yes	174
10	2,019,009	F	28	Respiratory diphtheria	Miranda	*Corynebacterium diphtheriae*	mitis	Yes	Yes	174

*ND* not defined, *NA* not applicable, *ND* no determined.

### Antimicrobial characteristics

All *C. diphtheriae* (n = 8) and *C. ulcerans* (n = 1) isolates were susceptible to erythromycin and penicillin.

### Multilocus sequence typing

Two STs were identified among the 8 *C. diphtheriae* analysed. The ST174 with 4 (50%) isolates and the ST697 with 4 (50%) isolates from Carabobo. The ST697 was not previously described. Clonal analysis classified the 8 *C. diphtheriae* isolates into one clonal complex designated by eBurst groups. The isolates are part of eBurst group 33 (composed of ST697, ST174 and ST677).

The *C. ulcerans* isolate belonged to a new ST (ST702) classified as a singleton by eBurst analysis.

### Whole genome sequencing results

Cg-MLST with an in-house generated scheme, consisting of 1441 *C. diphtheriae-*specific target loci, and visualization in a minimum spanning tree revealed only one cluster, consisting of four isolates from Carabobo (ST697). The differences between the isolates within the cluster ranged from zero to five alleles. The other four isolates were not assigned to any clusters and the differences between them ranged from 10 to 18 alleles. The reference sequence BH8NC_016800 (BioProject: PRJNA224116 and BioSample: SAMN02603077) from Brazil, harbours the lowest number of allele differences (1100 alleles) compared to the Venezuelan isolates (Fig. 4). The cg-MLST scheme was enlarged by adding 735 accessory targets to the 1441 cg-MLST targets. The results using the extended cg-MLST confirmed the phylogenetic structure.

## Discussion

This study shows a non-previously described sequencing type of *C. diphtheriae* implicated in a diphtheria outbreak and suggests its potential coexistence with *C. ulcerans* in the Venezuelan outbreak. Also, our findings contribute to shed light in describing a territorial pathway of this epidemic.

Some characteristics of the cases prospectively recruited for this study are similar to those retrieved from the hospital records and those officially reported by PAHO. Consistently, the most affected group of individuals was in the ages between 10 and 19 years^10^. In both, PAHO reports and the retrieved hospital records, individuals between 40 to 49 years of age were also frequently affected^10^. The high incidence and CFR in the latter group is an unusual finding that requires closer attention, since it might imply the potential need of vaccine boosters among non-pediatric population. Unfortunately, this assumption can only be made in the case these group was previously fully vaccinated as children (6-doses scheme^23^). Although likely since no coverage problems were reported in the country in the past decades, this information is missing for our cohort.

High incidences and CFR in diphtheria epidemics are expected among children under five^3^. This was also found during the latest outbreak among the Rohingya refugees and in the last outbreaks in Laos and in Yemen^3,6,7,24^. In the study presented here, it could be confirmed that patients under 5 years of age had the highest CFR followed by adults between 40 to 49 years. Although most of the deaths in this age group were seen in Carabobo (the state of highest incidence), sufficient clinical information is lacking to draw further conclusions. The overall CFR of 21% found in the study was high in comparison with the last two largest outbreaks registered during crises, namely the Rohingya displacement in 2016^7^ and the civil war in Yemen between 2017 and 2018^5^ where CFRs of 1.0% and 5.6% were reported, respectively. A delay in antitoxin administration and use of suboptimal doses due to country-wide shortages might be one explanation of the high CFR reported in this study. In another cohort that was observed during the same outbreak, up to 39.6% of cases had an unknown immunization status while 37.5% declared incomplete or no vaccination. Further, the same study showed that suboptimal dosage was frequently administrated to patients with diphtheria^25^.

The occurrence of diphtheria outbreaks might indicate vaccination coverage problems. In order to prevent diphtheria, a critical vaccination coverage (Vc) between 79 and 84% is essential to ensures herd immunity^26^. Coinciding with PAHO, the data retrospectively retrieved from the hospitals for this study suggest the beginning of the outbreak in the southeast of the country^10^. This indicates that the potential index case(s) might have originated from inaccessible mining areas where vaccination is challenging. The state of Bolivar is the largest in the country and the richest in mineral residues. Since the government declared the unrestrained opening of the mining arch of the Orinoco River in 2011, legal and illegal mining activities are ongoing and rapidly growing. This situation has also led to an exceptional escalation of vector-borne disease transmission in this region^27,28^. The detection of diphtheria cases among nomad Amerindian communities surrounding the mining areas^9^ may explain the transmission from these remote areas to other states of the country which is also represented in our retrospective data (Fig. 3a,b). These communities typically lack access to formal health services and frequently take part in mining activities. However, no strains from these areas were isolated in our study. The retrospective records included in this study aligns with the official reports which might indicate that the outbreak started in the southeast of the country (Bolivar state) and spread to the west and central most densely populated states (Fig. 4)^10^. Colombia and secondly Brazil, are the most frequent receptors or transit countries of Venezuelan refugees. This migration route may explain the cases detected among Venezuelan refugees at border communities of Colombia^9^. Political instability and a deep economic crisis have produced an unprecedented social calamity, characterized by the health system breakdown and a humanitarian emergency obliging thousands to leave the country^9,27–29^.

The eight *C. diphtheriae* isolates analysed are distributed in two STs (ST174 and ST697). The ST174 has only been reported in the MLST database PubMLST.org in one isolate from Brazil in 2016 from a six years old child and was toxin positive by PCR (no information about ELEK test is available) allowing to suspect potential strain introduction through the southeast boarder to Brazil. All isolates belong to the same eBurst group (group 33 composed of ST174, ST697 and ST677). Interestingly, with entered date corresponding to the 23rd of March 2020, the ST677 is described in PubMLST.org in one isolate from Colombia.

The phylogenetic analysis of the isolates by cgMLST revealed only one cluster (corresponding with the isolates grouped in the ST697). The cluster is concentrated around a specific geographic area (Carabobo) suggesting no transmission from one city to another occurred. Two of these four isolates (2,018,020 and 2,018,021) had no allele differences and differed by only 1 or 5 alleles compared to the other two (2,017,025 and 2,018,023). Samples within and outside the cluster were taken in similar time intervals suggesting that the strains detected in the different states might correspond to different origins.

The cg-MLST pointed to a less close relationship for isolates with ST174 showing between 10 to 18 different alleles. Neither the ST174 nor the ST677 isolate reported in PubMLST.org from Brazil and Colombia were characterized by WGS. To our knowledge, no complete genomes (except the one used as reference genome) from Brazil or Colombia exist to analyze the similarity between the circulating Venezuelan strains. However, our finding suggest that there may be a diverse community of corynebacterial strains circulating in South America.

Interestingly, the isolation of *C. ulcerans* in one patient with typical clinical features of diphtheria suggest the coexistence of both *C. ulcerans* and *C. diphtheriae* in the area of the outbreak. A publication by Dias and colleagues demonstrated the presence of this zoonotic pathogen not only in rural areas of Brazil but also in highly populated cities like Rio de Janeiro^30^. Its potential to generate outbreaks in humans should not be underestimated since a diverse diphtheria toxin in *C. ulcerans* has been identified^31^. The diphtheria toxin diversification might decrease the effectiveness of the diphtheria toxoid vaccine and the diphtheria antitoxin for preventing and treating infections caused by this bacteria.

A main limitation of this study was access to comprehensive data. Data on vaccination status as well as clinical information and treatment details are key to describe the nature and virulence of the circulating strains as well as the efficacy and coverage with the available vaccines. Although CFRs from our cohort is consistent with the reported by PAHO, our data collected in referral hospitals alone might not accurately represent the totality of cases. The lacking laboratory capacities for the confirmation of suspected cases, and the logistic challenges for timely shipping viable samples within Venezuela and to Madrid was a significant restriction and likely played a role in the low detection rate of our cohort mostly compounded by highly suspected cases of diphtheria. However, to our knowledge, this is the only study of prospective cases accompanied with retrospective medical records together with molecular analysis of bacterial strains implicated in this large outbreak. Offering an opportunity to spread crucial data of a remerging disease in a context of extreme logistic difficulties and political arrest.

## Conclusions

The diphtheria outbreak in Venezuela might remain active up to date, reflecting the inadequacy of implemented outbreak mitigation strategies. The high CFR observed in the study population may be related to the delay in antitoxin administration and the use of suboptimal doses. The unexpectedly high frequency in adolescents and adults suggests the need for vaccine booster doses among the non-pediatric population. The WGS analysis revealed that the outbreak seems to be caused by different strains with *C. diphtheriae* and *C. ulcerans* coexisting.
